# Association between Serum Chemerin Concentrations and Clinical Indices in Obesity or Metabolic Syndrome: A Meta-Analysis

**DOI:** 10.1371/journal.pone.0113915

**Published:** 2014-12-03

**Authors:** Ya Li, Bingyin Shi, Sheli Li

**Affiliations:** 1 Department of endocrinology, The First Hospital Affiliated to Xi'an Jiaotong University, Xi'an 710061, China; 2 Department of endocrinology, Yan'an University Affiliated Hospital, Yan'an 716000, China; University of Modena & Reggio Emilia, Italy

## Abstract

**Objective:**

Chemerin is a novel adipokine. Previous research has investigated the association between chemerin and clinical indices in patients with obesity or metabolic syndrome (MS), although the results obtained have been inconsistent. We conducted a meta-analysis to investigate the association between chemerin and clinical indicators of diabetes, MS and obesity with obesity or MS subjects.

**Design and Methods:**

Studies were identified by searching the PubMed, the Cochrane Library, EMBASE and CNKI, databases beginning with the original report in July 2007 until the end of May 2013. For each variable, summary correlation coefficients were estimated using random-effects or fixed-effect meta-analysis with 95% confidence interval (CI) performed by STATA software.

**Results:**

A total of eight studies with 20 clinical variables (total n = 1787) met the inclusion criteria. The meta-analyse of diabetes markers showed that FSI (*r_s_* = 0.26; 95% CI = 0.21–0.31; P = 0.000), 2HPG (*r_s_* = 0.06; 95% CI = 0.01–0.12; P = 0.030) and HOMA-IR (*r_s_* = 0.178; 95% CI = 0.019–0.337; P = 0.028) were positively correlated with chemerin, however, FPG (*r_s_* = 0.03, 95% CI = −0.02 to 0.08, P = 0.240) and HbA1c (*r_s_* = −0.05; 95% CI = −0.24–0.15; P = 0.641) were not significantly correlated with chemerin. The meta-analyses of MS and obesity markers indicated that TG, TC, CRP BMI, TBF%, WC, WHR and Leptin were positively correlated with chemerin, nevertheless, SBP, DBP, LDL-C, HDL-C, ALT and r-GT were not significantly correlated, adiponectin was negatively correlated. Sensitivity analysis was performed and the summary results did not change significantly.

**Conclusions:**

The results suggest that chemerin in patients with obesity or MS may be associated with obesity, imbalances in lipid and diabetes metabolism and insulin resistance. Chemerin played an important role in the pathophysiology of obesity and MS.

## Introduction

With the change of modem lifestyle and the improvement of living standards, the prevalence of metabolic syndrome is increasing fast. Metabolic syndrome(MS), a series of clinical symptoms, which combined with metabolic diseases, seriously affects human health. The major clinical changes of MS include central obesity, diabetes or sugar damage, hypertension, and dyslipidemia.Insulin resistance is a common pathophysiological basis for MS [1].

Chemerin, a recently discovered adipocytokine, is a chemoattractant protein that acts as a ligand for the G protein-coupled receptor CMKLR1 (also known as ChemR23) [2]. Chemerin is a 14 kDa protein secreted in an inactive form as prochemerin and is activated through cleavage of the C-terminus by inflammatory and coagulation serine proteases [3]. Chemerin has been implicated in autocrine/paracrine signaling for adipocyte differentiation and also stimulation of lipolysis [4]. It has been shown to regulate adipocyte differentiation and modulate the expression of adipocyte genes involved in glucose and lipid homeostasis, such as glucose transporter-4, adiponectin and leptin [5].

A study by Fatima et al. [6] indicated that circulating chemerin levels were significantly higher in obese subjects with BMI greater than 25 kg/m^2^ compared with those with a BMI below 25 kg/m^2^. In morbidly obese patients undergoing bariatric surgery, sustained reduction of chemerin serum concentrations was associated with weight loss and improvement of metabolic parameters [7], [8]. In human subjects, circulating chemerin was shown to be strongly associated with multiple components of metabolic syndrome, including body mass index (BMI), triglycerides, high-density lipoprotein cholesterol (HDL-C) and hypertension [4], [7], [9], it was associated with systemic markers of inflammation, such as high-sensitivity C-reactive protein (hsCRP), interleukin-6 (IL-6) and tumor necrosis factor-α (TNF-α) [10]–[12]. However, the correlation analyses of Alfadda et al. [13] showed that no correlation was found between serum chemerin concentrations and fasting glucose, total cholesterol, low density lipoprotein cholesterol (LDL-C), triglycerides, insulin, C-reactive protein or adiponectin. In addition, there are many inconsistencies in the research results of correlation analyses [14].

To our knowledge, although many studies (approximately133) have been published on serum chemerin levels in patients with obesity or MS, a meta-analysis has not yet been performed aiming to integrate the findings of individual studies that investigated relationships between chemerin and various clinical indices in obesity or MS. The present meta-analysis aimed to systematically examine the currently available evidence on the associations of chemerin concentrations and clinical indices and to determine the associations between serumchemerin concentrations and indices of diabetes, MS and obesity in subjects with obesity or MS.

## Materials and Methods

### Literature search and study selection

Studies were identified by searching the PubMed, the Cochrane Library, EMBASE and China national knowledge infrastructure (CNKI) databases beginning with the original report in July 2007 [4] until the end of May 2013 [15] using the terms ‘chemerin’ and ‘obesity’ OR ‘fat’ OR ‘metabolic syndrome’ OR ‘MS.’ References of relevant studies were also screened for eligibility. Additionally, we manually retrieved or wrote to authors to ask for unpublished or more complete information. We selected English-language and Chinese-language, original research articles that included subjects with obesity or MS.

**Figure 1:**
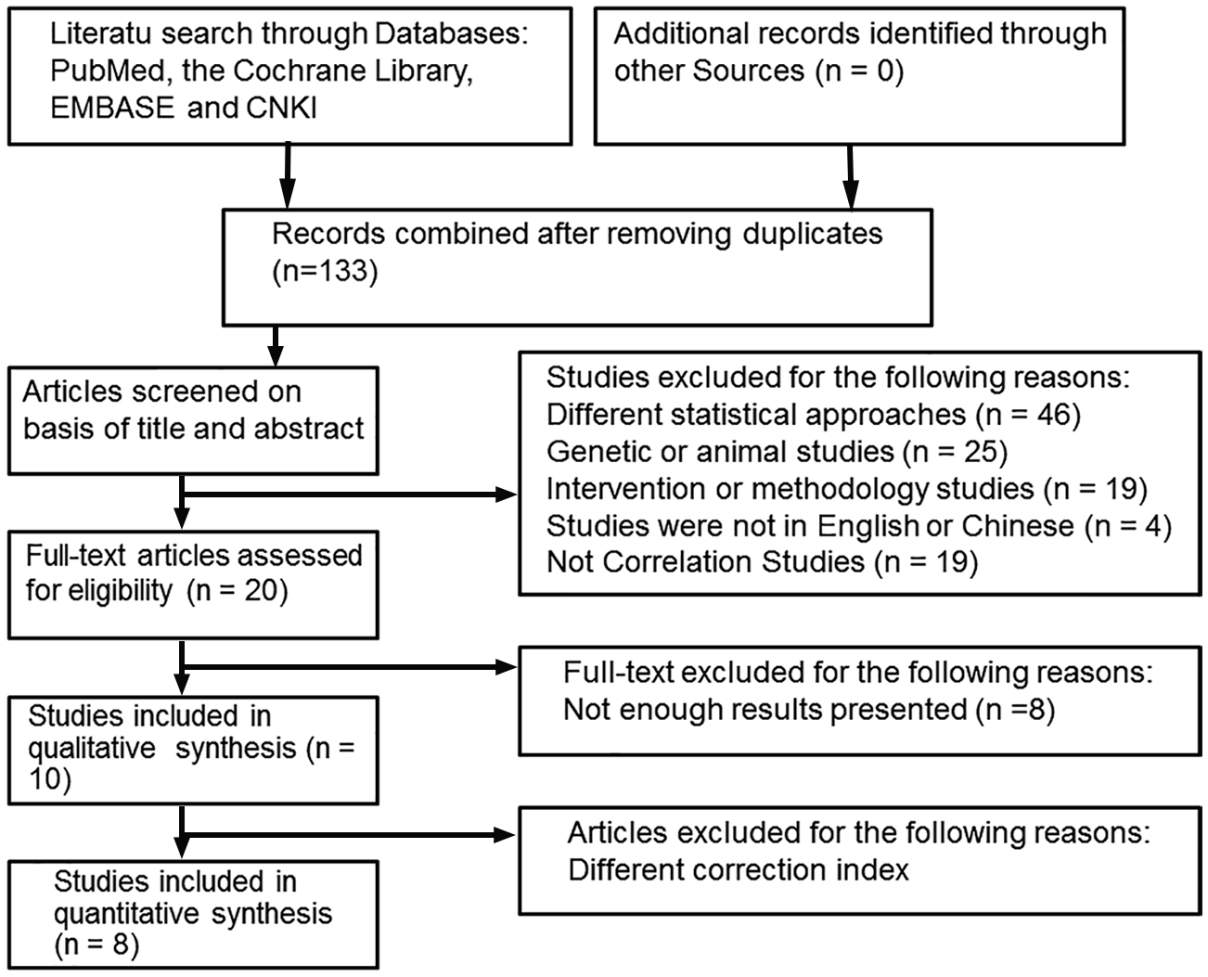
Selection process for studies included in the meta-analysis.

Studies were included if serum chemerin concentrations and clinical variables had been measured in human subjects with obesity or MS and if they included linear regression coefficients or correlation coefficients quantifying the relationship between serum Chemerin concentrations and the measured clinical variables and if age and gender were adjusted on the linear regression analysis. Studies were excluded if only as abstracts, reviews, duplicate publications of the same dataset or non-original reports (unless including new data). Ten studies met the inclusion criteria: [4], [6], [9], [12]–[18]. Two of the studies were later excluded because Age and BMI were adjusted on the linear regression analysis [6], [18]. Therefore, a total of eight independent studies were included in the meta-analyses (Fig. 1).

### Data extraction

We produced a data extraction form to assemble previously defined relevant information from the studies, including: a. the general information of included studies (e.g. number, publication year, country, etc.); b. study characteristics (e.g. sample size, craft, etc.); c. correlation coefficient(e.g. Pearson correlation coefficient and Spearman correlation coefficient, etc.); d. Diabetes markers (e.g. FPG, FSI,HOMA-IR,HbA1c, etc.); e. Obesity markers (e.g. BMI, WHR, Adiponectin, Leptin, etc.); f. Metabolic syndrome markers (e.g. SBP, LDL-C, CRP, r-GT, etc.) Data extraction was performed independently by two investigators. If disagreement occurs, an independent investigator would be asked to help reach agreement.

### Data synthesis and statistical analysis

Spearman correlation coefficients were used for meta-analyses of data that had been log transformed before analysis, because they are unaffected by monotonic transformations such as the logarithmic transformation. Therefore the use of Spearman correlation coefficients allowed us to include the maximum number of studies in the meta-analyses. When necessary, published Pearson correlation coefficients were converted to Spearman correlation coefficients using equation (1), which expresses the asymptotic relationship between Pearson and Spearman correlation coefficients: 
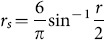
(1)where *r_s_* is the Spearman correlation coefficient and r is the Pearson correlation coefficient [19].

Spearman correlation coefficient sampling distribution standard error (SE) depends on the value of the correlation coefficient. This is problematic so, the Fisher transformation shown in equation (2) was used to convert each correlation coefficient to an approximately normally distributed variable z with SE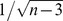, where *n* is the sample size: 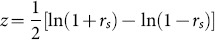
(2)


Appropriately converted data from the studies were combined using random effects meta-analyses [20]. The Fisher-transformed data were converted back to the original scale to enable the data to be plotted and interpreted, by inverse transformation shown in equation (3):
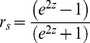
(3)


Forest plots were constructed to show the 95% confidence intervals (CIs) for the correlation coefficients from each of the included studies and the combined correlation coefficient for each meta-analysis. Heterogeneity between studies was assessed by the I^2^ statistic, which represents the amount of the total variation that can be explained by between-study variation [21], [22]. I^2^ values of approximately 25–50%, are considered indicative of low, 50–75% moderate and ≥75% of high heterogeneity. A random effects model was performed if significant heterogeneity (I^2^>50%, p<0.1) was observed between studies; Otherwise, a fixed effects model was adopted (I^2^<50%, p>0.1) [21], [22]. Sensitivity analysis was performed by removing each study in the meta analysis one at a time to detect its influence on pooled OR. We investigated the potential sources of heterogeneity by meta-regression analysis. All statistical analyses were performed using Stata/SE, version 12 (Stata Corporation, College Station, TX, USA).

## Results

### Study characteristics

Eight studies were identified and included in the analysis (Table 1). Two of the studies were performed in America (n = 1014) [9], [17], one of them is Mexican - American (n = 969) [9]. Two of the studies were performed in Korea (n = 1 27) [15], [16], one in China (n = 76) [14], one in Mauritius (n = 142) [4], one in Germany (n = 303) [12] and one in Saudi Arabia (n = 125) [13]. Of the included studies, five studies reported the Spearman correlation coefficient of chemerin and markers [9], [12], [14], [15], [17], two studies informed the Pearson correlation coefficient [13], [16] and one reported Spearman correlation coefficient or Pearson correlation coefficient [4].

**Table 1:** Characteristics of the studies included in the meta-analyses.

	First author	Bozaoglu	Wang	Bozaoglu	Jialal	Chu	Lehrke	Kim	Alfadda
	Year	2009	2009	2007	2013	2012	2009	2013	2012
	study population (n)	Mexican-American \ MS(969)	China/Obesity (76)	Mauritius/Obesity OR MS(142)	America/MS (45)	Korea \ MS (92)	Germany \ MS (303)	Korea/Obesity (35)	Saudi Arabia/Obesity (125)
Correlation	Coefficient	S	S	S OR P	S	P	S	S	P
Diabetes markers	FPG	X	X	X		X		X	X
	2HPG	X	X	X		X		X	
	FSI	X	X	X		X		X	X
	HOMA-IR	X	X	X	X	X		X	X
	HbA1c		X					X	
Obesity markers	BMI	X	X	X		X	X	X	
	TBF%	X		X		X		X	
	WC		X			X		X	
	WHR		X	X				X	
	Adiponectin					X	X	X	X
	Leptin						X		X
Metabolic syndrome markers	SBP	X	X	X	X	X		X	
	DBP	X	X	X		X		X	
	LDL-C		X				X		X
	HDL-C	X	X	X	X	X	X	X	X
	TG	X	X	X	X	X	X	X	X
	TC	X	X	X		X		X	X
	CRP	X			X	X	X	X	X
	ALT		X					X	
	r-GT		X					X	

ALT, Alanine Aminotransferase; BMI, body mass index; CRP, C-reactive protein; DBP, diastolic blood pressure; FSI, fasting serum insulin; FPG, fasting plasma glucose; r-GT, r-Glutamyl Transpeptidase; HbA1c, hemoglobin A1c; HDL-C, high-density lipoprotein cholesterol; HOMA-IR, homeostasis model of assessment for insulin resistence index; 2HPG, 2H postprandial plasma glucose; LDL-C, low-density lipoprotein cholesterol; P, Pearson correlation coefficient; S, Spearman correlation coefficient; SBP, systolic blood pressure; TC, total cholesterol; TG, triglyceride; TBF%, total body fat (%); WC, waist circumference; WHR, waist-hip ratio.

### Diabetes markers and chemerin

**Figure 2:**
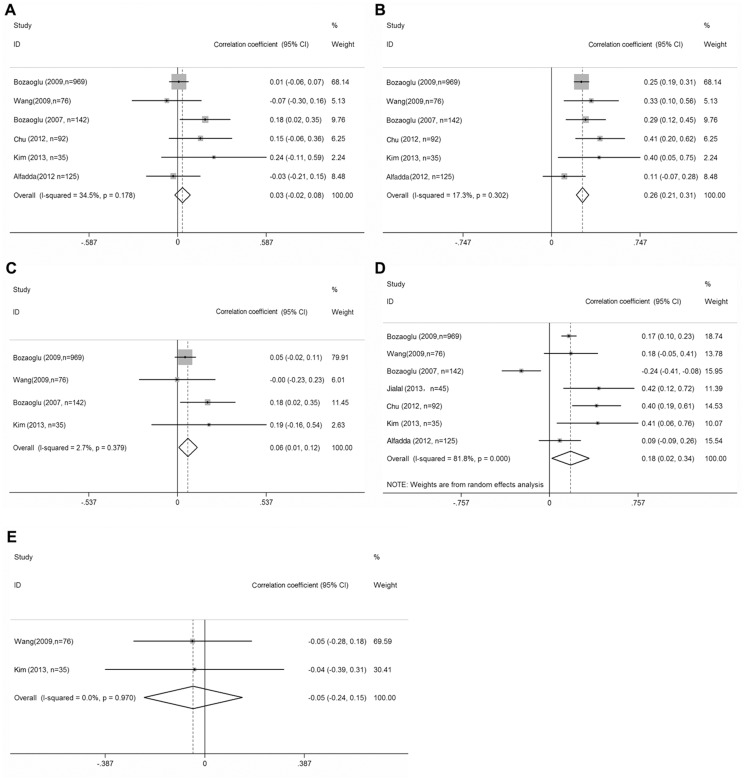
Correlations between serum chemerin concentrations and diabetes markers in Obesity or MS subjects. Summaries are shown of the correlations between serum chemerin concentrations and (a) fasting plasma glucose concentrations, (b) fasting serum insulin concentrations, (c) 2H postprandial plasma glucose, (d) HOMA_IR and (e) Hemoglobin A1c. 95% confidence intervals (CIs) are represented by the horizontal lines, and diamonds represent the overall estimate and 95% CI.

Six of the studies presented data on the association between FPG and chemerin concentrations in patients with obesity or MS (total n = 1439; Fig. 2a) [4], [9], [13]–[16]. Fig. 2a (using fixed-effects model) showed these five markers were not significantly correlated with serum chemerin concentrations, nor was the overall correlation coefficient statistically significant (*r_s_* = 0.03, 95% CI–0.02 to 0.08, P = 0.240). Six researches examined the association between FSI and serum chemerin concentrations in patients with obesity or MS (total n = 1439; Fig. 2b) [4], [9], [13]–[16]. Four studies investigated the association between 2HPG and serum chemerin concentrations (total n = 1222; Fig. 2c) [4], [9], [14], [15]. Eventually, FSI (*r_s_* = 0.26; 95% CI = 0.21–0.31; P = 0.000) and 2HPG (*r_s_* = 0.06; 95% CI = 0.01–0.12; P = 0.030) were positively correlated with serum chemerin concentrations. Seven studies examined the association between HOMA-IR and serum chemerin concentrations (total n = 1484; Fig. 2d) [4], [9], [13]–[17]. The Fig. 2d (using random-effects model) suggested that HOMA-IR was positively correlated with serum chemerin concentrations (*r_s_* = 0.178; 95% CI = 0.019–0.337; P = 0.028). Based on sensitivity analysis, the study on the maximum of heterogeneity was excluded [9]; HOMA-IR resulted in a summary coefficient of 0.233 (95% CI = 0.126 to 0.341; P = 0.000). Two researches investigated the association between HbA1c and serum chemerin concentrations (total n = 1222; Fig. 2e) [14], [15]. On the whole, HbA1c was not correlated with serum chemerin concentrations (*r_s_* = −0.05; 95% CI = −0.24–0.15; P = 0.641).

### Metabolic syndrome markers and chemerin

**Figure 3:**
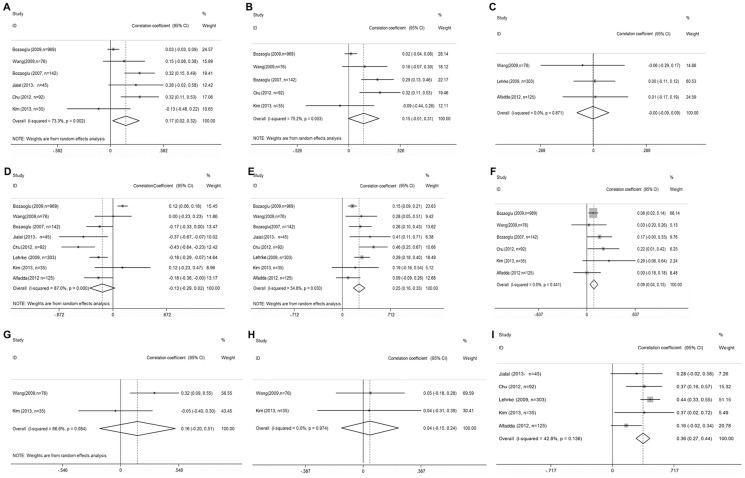
Correlations between serum chemerin concentrations and metabolic syndrome markers in Obesity or MS subjects. Summaries are shown of the correlations between chemerin concentrations and (a) systolic blood pressure, (b) diastolic blood pressure, (c) low-density lipoprotein cholesterol, (d) high-density lipoprotein cholesterol, (e) triglyceride, (f) total cholesterol, (g) Alanine Aminotransferase, (h) r-Glutamyl Transpeptidase and (I) C-reactive protein. 95% confidence intervals (CIs) are represented by the horizontal lines, and diamonds represent the overall estimate and 95% CI.

The TG (total n = 1787; Fig. 3e) and HDL (HDL; total n = 1787; Fig. 3d) were measured in all eight studies [4], [9], [12]–[17]. Overall, TG (*r_s_* = 0.25; 95% CI = −0.16–0.33; P = 0.030) was positively correlated with serum chemerin concentrations, whereas HDL was not significantly correlated (*r_s_* = −0.134; 95% CI = −0.291–0.024; P = 0.097). TC (total n = 1439; Fig. 3f) was analyzed in six studies [4], [9], [13]–[16] and LDL (total n = 504; Fig. 3c) were measured in three studies [12]–[14]. TC (*r_s_* = 0.093; 95% CI = 0.041–0.145; P = 0.000) was positively correlated with serum chemerin concentrations, whereas LDL were not significantly correlated with serum chemerin concentrations (*r_s_* = −0.003; 95% CI = −0.092–0.085; P = 0.939). TG was more strongly correlated with serum chemerin concentrations than TC. ALT and r-GT (total n = 111; Fig. 3g, 3h) were measured in two studies [14], [15]. Overall, ALT and r-GT were not significantly correlated with serum chemerin concentrations 0.158 (95% CI = −0.199–0.514; P = 0.387); −0.045 (95% CI = −0.146–0.236; P = 0.646). SBP (total n = 1359; Fig. 3a) was measured in six studies [4], [9], [14]–[17] and DBP (total n = 1314; Fig. 3b) was analyzed in five studies [4], [9], [14]–[16]. SBP (*r_s_* = −0.170; 95% CI = 0.022–0.317; P = 0.025) was positively correlated with serum chemerin concentrations, whereas DBP was not significantly correlated (*r_s_* = −0.151; 95% CI = −0.006–0.309; P = 0.059). Five studies investigated the association between CRP and serum chemerin concentrations (total n = 600; Fig. 3i) [12], [13], [15]–[17], CRP was positively correlated with serum chemerin concentrations (*r_s_* = 0.355; 95% CI = 0.274–0.436; P = 0.000).

### Obesity markers and chemerin

**Figure 4:**
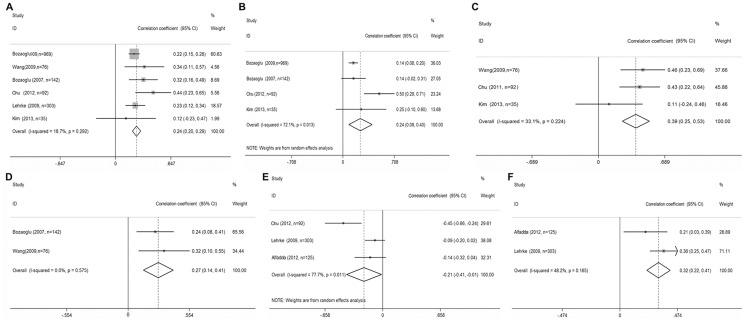
Correlations between serum chemerin concentrations and obesity markers in Obesity or MS subjects. Summaries are shown of the correlations between chemerin concentrations and (a) body mass index, (b) total body fat (%), (c) waist circumference, (d) waist-hip ratio, (e) Adiponectin and (f) Leptin. 95% confidence intervals (CIs) are represented by the horizontal lines, and diamonds represent the overall estimate and 95% CI.

Six studies investigated the association between BMI and serum chemerin concentrations in patients with obesity or MS (total n = 1617; Fig. 4a) [4], [9], [12], [13], [15], [16], TBF% (total n = 1238; Fig. 4b) was measured in four studies [4], [9], [15], [16], BMI (*r_s_* = 0.245; 95% CI = 0.196–0.295; P = 0.000) and TBF% (*r_s_* = 0.241; 95% CI = 0.081–0.401; P = 0.003) were positively correlated with serum chemerin concentrations. WC (total n = 203; Fig. 4c) and WHR (total n = 218; Fig. 4d) were each measured in studies (WC: [14]–[16], WHR: [4], [14]). Both showed a positive correlation with chemerin concentrations. Summary correlation coefficients were 0.388 (95% CI = 0.2473–0.528; P = 0.000) and 0.272 (95% CI = 0.137–0.406; P = 0.000), respectively. Adiponectin and leptin concentrations were analyzed in some studies (Adiponectin: [12], [13], [16]; leptin: [12], [14]. Leptin was positively correlated with serum chemerin concentrations (*r_s_* = 0.317; 95% CI = 0.221–0.413; P = 0.000; total n = 428; Fig. 4f), whereas adiponectin was negatively correlated with serum chemerin concentrations (*r_s_* = −0.214; 95% CI = −0.414–0.013; P = 0.037; total n = 520; Fig. 4e).

### Sensitivity analysis

Sensitivity analysis was performed by the sequential removal of individual studies and cumulative statistics for all comparisons of all subjects showed that the summary results did not change significantly, indicating good stability of the included studies.

### Test of heterogeneity

The heterogeneity was existent. Therefore, we performed a meta-regression analysis to identify the sources of heterogeneity by country, correlation coefficient(S OR P)and experimental group subjects(Obesity or MS). However, when we categorized the heterogeneity by these factors none significantly contributed to the observed heterogeneity.

## Discussion

In order to address any association of serum chemerin concentrations with diabetes, metabolic syndrome and obesity in obesity or MS subjects, we performed meta-analysis of the published literature. Eight studies of high quality were selected from a larger sample identified. Parameters of diabetes, MS and obesity were examined after adjustment for age and gender. A significant correlation between obesity parameters and serum chemerin concentrations in patients with obesity or MS were affirmed by meta-analysis. Among diabetes and MS parameters, meta-analyses confirmed a strong correlation between several diabetic and lipid metabolism markers and serum chemerin concentrations in patients with obesity or MS.

In obesity, transformed secretion of various adipokines is closely linked to metabolic changes that eventually result in associated metabolic diseases. Several studies have affirmed that circulating chemerin levels are boosted in both obese humans and obese/diabetic experimental animals and are positively correlated with various aspects of MS [4], [23], [24]. Similar to previous reports, we found a positive, statistically significant correlation between circulating chemerin and BMI. In contrast to these same studies, we found no association between circulating chemerin and insulin resistance markers [4], [23], [24]. Nevertheless, the meta-analysis approve that BMI and HOMA-IR are positively correlated with serum chemerin concentrations.

As a new kind of fat cytokine and chemokine, chemerin had been demonstrated to be strongly associated with obesity [9]. The mechanisms of chemerin in obesity might be as follows. Firstly, chemerin could promote the differentiation of adipocytes and metabolism. Secondly, chemerin could inhibit the decomposition of fat to a certain extent. Thirdly, chemerin might participate in the inflammatory immune response. Obesity could cause inflammation of adipose tissue, and serum chemerin has characteristics of a recruited and activated immune cell [25]. It could be presumed that serum chemerin might participate in adipose tissue inflammation in obese individuals [26]. The research of Stejskal et al to in a caucasian population demonstrated that serum chemerin was correlated with WC and BMI after adjustment for age, gender, blood pressure, blood lipids, and was consistent with the results of meta-analyses. This suggests that serum chemerin is correlated with MS with central obesity as a characteristic, and central obesity may be a factor in the increased plasma chemerin levels [27].

However, it is controversial whether chemerin is related to T2DM in humans. It has been shown that chemerin in 3T3-L1 adipocytes improves insulin stimulated glucose uptake through the insulin signaling. This suggested that chemerin may regulate insulin sensitivity of adipose tissue [28], In that case with increase in chemerin concentration, the concentration of insulin should decline, but the experiment showed, FSI were positively correlated with serum chemerin. This result may be because that it needed more chemerin to regulate insulin sensitivity in the pathological state of diabetes and insulin resistance, so the mechanism of the relationship between chemerin and insulin, still needs further exploration.

In addition, Takahashi M et al. approved the involvement of chemerin levels in sex dimorphism, which indicated that the serum chemerin levels in males were higher than those in females in a Japanese population [29]. In contrast, Bozaoglu et al.demonstrated that the serum chemerin levels were significantly higher in females compared with males in a Mexican-American population [9]. Stejskal et al. showed that no significant difference was observed in the serum chemerin levels between sexes in 55 non-obese Caucacian control subjects [27]. The reason was inferred that the racial difference or the difference in the background of population, i.e. BMI might affect the results, and sex hormones might regulate serum chemerin concentration. Furthermore, aging is known to be associated with increased weight, insulin resistance, and adiposity gain. Aging in T2DM patients is associated with an altered chemerin secretion [30]. Consequently, the results of meta-analysis are more reliable after adjustment for age and gender.

### Heterogeneity

Previous study declared that it was difficult to avoid heterogeneity in meta-analysis [31]. With regard to our studies, we mentioned several problems that may be responsible for heterogeneity: a. Different geographical locations of the included studies: two studies were from America, two studies were from Korea, while others were from China, Mauritius, Germany and Saudi Arabia respectively; b. Correlation coefficient: five studies reported the Spearman correlation coefficient, two studies reported the Pearson correlation coefficient and one reported Spearman or Pearson correlation coefficient, all Pearson correlation coefficients were converted to Spearman correlation coefficients using equation; c. Different sample sizes: one of the included studies [9] had largest sample sizes with 969 subjects, another had smallest sample sizes with 35 subjects [15].

### Limitations

Results from our meta-analysis must be viewed cautiously due to its own limitations. Firstly, we only selected English and Chinese published or unpublished literature, thus linguistic bias exists. Secondly, given the limitation of local libraries we only selected the literature for which original full-text was likely to be found, which would cause selection bias. Thirdly, there were publication bias and large statistical heterogeneity could be found among our included studies, which might lead to the existence of bias factors. In spite of only identifying eight studies for meta-analysis which used unequal sample sizes and different subjects of experimental groups; as the original data were not available for further analysis, the results should be interpreted with caution. Although these limitations, we have tried to moderate and explain them.

## Conclusions

For the present meta-analyses, 20 clinical indices in three categories (related to diabetes, MS and obesity) were individually analysed for correlation with serum chemerin concentrations in patients with obesity or MS. The meta-analyses showed that all obesity markers, some MS and diabetes markers were significantly correlated with serum chemerin concentrations, whereas few MS and diabetes markers were not. chemerin concentrations played an important role in the pathophysiology of obesity and metabolic syndrome.

## Supporting Information

Checklist S1 **PRISMA checklist.**
(DOC)
